# Viral Protein Kinetics of Piscine Orthoreovirus Infection in Atlantic Salmon Blood Cells

**DOI:** 10.3390/v9030049

**Published:** 2017-03-18

**Authors:** Hanne Merethe Haatveit, Øystein Wessel, Turhan Markussen, Morten Lund, Bernd Thiede, Ingvild Berg Nyman, Stine Braaen, Maria Krudtaa Dahle, Espen Rimstad

**Affiliations:** 1 Department of Food Safety and Infectious Biology, Faculty of Veterinary Medicine, Norwegian University of Life Sciences, 0454 Oslo, Norway; hanne.merethe.haatveit@nmbu.no (H.M.H.); oystein.wessel@nmbu.no (Ø.W.); turhan.markussen@nmbu.no (T.M.); ingvild.nyman@nmbu.no (I.B.N.); stine.braaen@nmbu.no (S.B.); 2 Department of Immunology, Norwegian Veterinary Institute, 0454 Oslo, Norway; morten.lund@vetinst.no (M.L.); maria.dahle@vetinst.no (M.K.D.); 3 Department of Biosciences, University of Oslo, 0316 Oslo, Norway; bernd.thiede@ibv.uio.no

**Keywords:** *Piscine orthoreovirus*, PRV, non-structural protein, µNS, µ1, expression kinetics, proteolytic cleavage, pathogenesis, blood cells, Atlantic Salmon

## Abstract

*Piscine orthoreovirus* (PRV) is ubiquitous in farmed Atlantic salmon (*Salmo salar*) and the cause of heart and skeletal muscle inflammation. Erythrocytes are important target cells for PRV. We have investigated the kinetics of PRV infection in salmon blood cells. The findings indicate that PRV causes an acute infection of blood cells lasting 1–2 weeks, before it subsides into persistence. A high production of viral proteins occurred initially in the acute phase which significantly correlated with antiviral gene transcription. Globular viral factories organized by the non-structural protein µNS were also observed initially, but were not evident at later stages. Interactions between µNS and the PRV structural proteins λ1, µ1, σ1 and σ3 were demonstrated. Different size variants of µNS and the outer capsid protein µ1 appeared at specific time points during infection. Maximal viral protein load was observed five weeks post cohabitant challenge and was undetectable from seven weeks post challenge. In contrast, viral RNA at a high level could be detected throughout the eight-week trial. A proteolytic cleavage fragment of the µ1 protein was the only viral protein detectable after seven weeks post challenge, indicating that this µ1 fragment may be involved in the mechanisms of persistent infection.

## 1. Introduction

*Piscine orthoreovirus* (PRV) belongs to the genus *Orthoreovirus* in the family *Reoviridae* [1,2]. The orthoreoviruses are ubiquitous in various animal species, but only found to be of pathogenic significance in poultry and recently in fish [3,4,5,6,7]. PRV is abundant in farmed Atlantic salmon (*Salmo salar*), detected both in apparently healthy and diseased fish [8,9,10,11]. The infection causes heart and skeletal muscle inflammation (HSMI) and is associated with melanised foci in white muscle in Atlantic salmon [1,7,12]. HSMI is a prevalent disease and melanised foci is a quality problem; both conditions are of major economic importance to salmon aquaculture. The pathogenesis of HSMI is not completely elucidated. Outbreaks of the disease are primarily observed in the seawater phase and last for several weeks in the population [13], after which the PRV infection becomes persistent [9,11,14]. In experimental cohabitant infection trials, disease onset occurs after 8–10 weeks [15].

The study of molecular mechanisms linked to PRV infection has been limited by the lack of susceptible cell lines. Studies of the viral infection have therefore been performed in vivo or by infecting erythrocytes ex vivo [16]. Piscine erythrocytes are nucleated and contain the transcriptional and translational machinery necessary for expression of mRNA and proteins [17]. Erythrocytes are important target cells for PRV and the infection activates an innate antiviral immune response typical for RNA viruses in these cells [18]. During the peak phase of infection, more than 50% of all erythrocytes may be infected [19]. Interestingly, severe anemia has not been reported from HSMI outbreaks in the seawater phase, indicating low or no virus-induced lysis of infected erythrocytes [20]. Recently, a variant of PRV was demonstrated to be the etiologic agent of erythrocytic inclusion body syndrome (EIBS), a condition associated with anemia and mass mortality in juvenile Coho salmon (*Onchorhynchus kisutchi*). The level of anemia in EIBS affected fish corresponded with the level of viral replication in blood [7]. In addition, infection of rainbow trout in fresh water by yet another PRV variant is also associated with anemia and an HSMI-like disease [5].

The *Orthoreovirus* genome consists of ten double-stranded RNA (dsRNA) segments enclosed in a double protein capsid. The genomic segments are classified according to size with three large (L), three medium (M) and four small (S) segments encoding the λ, µ and σ class proteins, respectively [3,21]. In mammalian orthoreovirus (MRV), the species type of genus *Orthoreovirus*, the viral transcription machinery is located in the inner core and consists of λ1, λ2, λ3, µ2 and σ2 [22]. The outer capsid proteins µ1, σ1 and σ3 are involved in cell attachment and membrane penetration during the initial stages of infection [23,24,25]. The two non-structural proteins µNS and σNS participate in the formation of viral factories where viral genome replication and particle assembly occur [21,26,27]. Although some important amino acid motifs are conserved between MRV and PRV, sequence identities between homologous proteins are generally low [2]. MRV enters the cell by receptor-mediated endocytosis. The outer capsid is largely removed and µ1 is cleaved at two positions that generate, in addition to the full-length protein, five different fragments [24,28]. The N-terminal autolytic cleavage site, which produces µ1N and µ1C, seems conserved across orthoreoviruses, including PRV [2,29,30]. Further cleavage of µ1C, mediated by exogenous proteases, generate fragments δ and φ [24].

Structures resembling viral factories have also been observed in PRV-infected erythrocytes, and recombinant expression of the protein in fish cell lines indicate that PRV µNS has an analogous role in factory formation [16,19,31]. The majority of virus-encoded proteins localize completely or partially within these viral factories [2,3,21]. The viral factories in PRV-infected cells resemble the globular structures observed for the MRV type 3 Dearing (T3D) strain, in contrast to the filamentous-like viral factories generated by MRV Type 1 Lang (T1L) [19]. The latter is considered the most common morphology type of orthoreoviral factories [21,32]. Gene segment M3 in MRV and avian orthoreovirus (ARV) are reported to produce two isoforms of the factory forming µNS protein in infected cells [33,34,35]. The second isoform is produced by different mechanisms in the two viruses; in MRV, µNSC is expressed by a second in-frame AUG (Met_41_) while in ARV, post-translational cleavage in the N-terminal region releases µNSN [33,35,36]. In ARV, only full-length µNS interacts with σNS in infected cells, suggesting that the two isoforms play different roles during ARV infection [34].

Considering the emerging occurrence of HSMI, PRV exhibits a considerable risk for the aquaculture industry and proper disease control is highly desired. To understand the association between PRV infection and disease outcome, and also to limit further disease outbreaks, more information regarding PRV protein kinetics is essential. In the present study, the kinetics of viral RNA, viral protein and antiviral immune response in blood cells from experimentally PRV-infected Atlantic salmon were investigated. We hypothesized that PRV causes an acute infection in blood cells correlating with innate antiviral gene expression, before the infection subsides to a low persistent level.

## 2. Materials and Methods

### 2.1. Construction and Expression of Recombinant *Piscine orthoreovirus* (PRV) μNS

Following the supplier’s protocol, the BaculoDirect™ Baculovirus Expression System (Invitrogen, Carlsbad, CA, USA) was used to generate recombinant µNS. The μNS open reading frame (ORF) (acc. no. KR337478) was obtained by polymerase chain reaction (PCR; primers listed in Table S1) of the plasmid construct pcDNA3.1 µNS N-FLAG [31] and cloned into the pENTR™ TOPO^®^ vector (Invitrogen). The pENTR µNS construct was used in a recombination reaction to generate the recombinant baculovirus DNA. Sanger sequencing (GATC Biotech AG, Konstanz, Germany) confirmed the sequence of the construct. Spodoptera frugiperda (Sf9) insect cells (BD Bioscience, Erembodegem, Belgium) cultured in Grace Insect Medium (Invitrogen) supplemented with 10% heat inactivated fetal bovine serum (FBS, Life Technologies, Paisley, Scotland, UK), 100 U/mL Penicillin, 100 µg/mL Streptomycin and 0.25 µg/mL Fungizone (Life Technologies), were transfected with recombinant baculovirus DNA. Passage 1 (P1) viral stock was harvested 11 days post transfection and used to produce high titer viral stocks according to the supplier’s protocol. The BacPAK quantitative PCR (qPCR) Titration kit (Clontech, Mountain View, CA, USA) was used to determine the viral titer. Finally, Sf9 insect cells were infected with Passage 2 (P2) or higher passage of recombinant baculovirus stock (>1 × 10^8^ copies/mL) and incubated at 27 °C for 96 h for expression of the recombinant µNS protein containing a C-terminal 6xHis-tag.

### 2.2. Construction and Expression of Recombinant PRV λ1

The ORF of PRV structural protein λ1 (acc. no. KR337475) encoded by gene segment L3 was amplified (primers listed in Table S1) using cDNA originating from a HSMI outbreak [31] as template. The PCR product was cloned into pET100/D-TOPO (Invitrogen) and the sequence verified by Sanger sequencing (GATC Biotech AG). The pET100-λ1 plasmid was transfected into *E. coli* (BL21 DE3 strain, Invitrogen) and expressed with a N-terminal 6xHis-tag, following the manufacturer’s instructions. Protein expression was monitored by sodium dodecyl sulfate polyacrylamide gel electrophoresis (SDS-PAGE).

### 2.3. Protein Purification

The Sf9 insect cells and the *E. coli* cells expressing recombinant PRV μNS and λ1 proteins, respectively, were pelleted by centrifugation at 5000× *g* for 10 min, then dissolved and washed in phosphate-buffered solution (PBS). Purification of recombinant proteins was carried out using ProBond Purification System (Life Technologies) following the manufacturer’s instructions. The recombinant µNS protein was eluted with an elution buffer containing 8 M Urea, 20 mM Na_2_H_2_PO_4_ (pH 4.0), and 500 mM NaCl. The purity of the recombinant protein was monitored by SDS-PAGE using a 4%–12% Bis–Tris Criterion XT gel (Bio-Rad, Hercules, CA, USA). To purify λ1, the Ni-NTA agarose was run on a SDS-PAGE where a band matching the size of λ1 was excised. The gel sample containing λ1 protein was solubilized in 250 mM Tris-HCl with 0.1% SDS, pH 6.8, sonicated 3 × 5 s and incubated at 4 °C with shaking overnight. The sample was centrifuged at 10,000× *g* for 10 min and the supernatant was dialyzed using the Slide-A-Lyser^®^ Dialysis cassette with 20,000 molecular weight cut-off (MWCO) and 0.5–3.0 mL capacity (Thermo Scientific, Waltham, MA, USA) following the manufacturer’s protocol. SDS-PAGE confirmed the purity of the recombinant λ1 protein. Protein concentrations for both μNS and λ1 were determined using the DC Protein Assay Reagent Package (Bio-Rad), with bovine serum albumin (BSA; Sigma-Aldrich, St. Louis, MO, USA) as protein standard.

### 2.4. Immunization of Rabbits

The purified recombinant proteins were used for immunization of rabbits and generation of antisera named anti-µNS #R320684 and anti-λ1 #K273. In the first injection, Freund’s complete adjuvant was added, thereafter the rabbits were boosted three times with Freund’s incomplete adjuvant weekly. The amount of µNS and λ1 antigen used per immunization was in the range of 45–500 μg. The rabbit sera produced were tested by Western blotting (WB) and fluorescent microscopy after transfection of epithelioma papulosum cyprini (EPC; ATCC CRL-2872) cells with pcDNA3.1 µNS N-FLAG [31] or pcDNA3.1 λ1 N-HA [31] (see description below). Antisera controls were collected prior to immunization. WB and immunofluorescent microscopy confirmed that the rabbit µNS and λ1 antisera recognized the µNS and λ1 proteins in transfected EPC cells (Figure S1). No staining was detected using the pre-immunization sera (data not shown).

### 2.5. Specificity of Antisera

EPC cells were cultivated in Leibovitz-15 medium (L15; Life Technologies) supplemented with 10% heat inactivated FBS, 2 mM l-glutamine, 0.04 mM mercaptoethanol and 0.05 mg/mL gentamycin-sulphate (Life Technologies), and seeded at a density of 1.5 × 10^4^ cells/well in a 24-well plate 24 h prior to transfection. Plasmids pcDNA3.1-µNS N-FLAG and pcDNA3.1-λ1 N-HA were transfected using Lipofectamine LTX reagent (Life Technologies) according to the manufacturer’s instructions. The cells were fixed and stained 48 h post-transfection with an Intracellular Fixation and Permeabilization Buffer Set (eBioscience, San Diego, CA, USA) following the manufacturer’s protocol. Antisera against µNS (1:1000) and λ1 (1:500); secondary antibody against rabbit IgG conjugated with Alexa Fluor 488 (Life Technologies) and Hoechst trihydrochloride trihydrate (Life Technologies) were used for staining. Images were captured on an inverted fluorescence microscope (Olympus IX81). Transfected EPC cells were also used to further verify anti-µNS and anti-λ1 in WB. A total of 3 × 5 million EPC cells were pelleted by centrifugation, resuspended in 100 μL Ingenio Electroporation Solution (Mirus, Madison, WI, USA) and transfected with 4 μg pcDNA3.1 µNS N-FLAG or pcDNA3.1 λ1 N-HA. The transfected cells were transferred to 75 cm^2^ culture flasks containing 20 mL pre-equilibrated L-15 growth medium (described above) and collected 72 h post-transfection. The cell pellets were lysed in Nonidet-P40 lysis buffer (1% NP-40, 50 mM Tris–HCl pH 8.0, 150 mM NaCl, 2 mM EDTA) containing Complete ultra mini protease inhibitor cocktail (Roche, Mannheim, Germany). The mix was incubated on ice for 30 min, and then centrifuged at 5000× *g* for 5 min at 4 °C. The supernatant was mixed with Sample Buffer (Bio-Rad) and Reducing Agent (Bio-Rad), denatured for 5 min at 95 °C and run in SDS-PAGE, using 4%–12% Bis–Tris Criterion XT gel (Bio-Rad). Magic Mark^TM^ XP Standard (Invitrogen) was used as a molecular size marker. Following SDS-PAGE, the proteins were blotted onto a polyvinylidene fluoride (PVDF) membrane (Bio-Rad) and anti-µNS and anti-λ1 were used as primary antibodies and anti-Rabbit IgG-HRP (GE Healthcare, Buchinghamshire, UK) as secondary antibody. Protein bands were detected by chemiluminescence (Amersham ECL Plus, GE Healthcare).

### 2.6. Experimental Challenge of Salmon

A cohabitation challenge experiment was performed at VESO Vikan aquatic research facility, (Vikan, Norway). The fish had an average weight of 30 grams at the onset of the experiment with a maximum stocking density of 80 kg/m^3^, and were kept in 0.4 m^3^ tanks supplied with filtered and UV-radiated fresh water, 12 °C ± 1 °C with a 12 h light/12 h dark regime. Water discharge of the tanks was provided by a tube overflow system with 7.2 L/min flow rate. The fish were acclimatized for two weeks prior to challenge, fed according to standard procedures and anesthetized by bath immersion (2–5 min) in benzocaine chloride (0.5 g/10 L water, Apotekproduksjon AS, Oslo, Norway) before handling. Briefly, the experimental study included one group of shedder fish (50%) marked at the time of PRV-injection by cutting off the adipose fin and one naïve cohabitant group (50%). The PRV inoculum was prepared from a batch of pooled heparinized blood samples from a previous PRV challenge experiment [19]. 

On day 0 of the challenge, the heparinized blood was diluted 1:2 in PBS and 0.1 mL of the inoculum was intraperitoneal (i.p.) injected into the shedders. The inoculum was confirmed negative for salmon viruses such as infectious pancreatic necrosis virus (IPNV), infectious salmon anemia virus (ISAV), salmonid alphavirus (SAV) and piscine myocarditis virus (PMCV) by reverse transcription quantitative PCR (RT-qPCR). Samples from six fish were collected before initiation of the experiment to provide time-0 uninfected control material for protein assays. Heparinized blood was collected from six cohabitant fish at each sampling point; 3, 4, 5, 6, 7 and 8 weeks post challenge (wpc). In addition, a second cohabitation challenge experiment lasting 10 weeks was performed at the same facility following a similar experimental design. In this study, six fish sampled prior to PRV challenge were used to provide uninfected control material for protein and RT-qPCR assays, and heparinized blood was collected from six cohabitant fish at 4, 6, 8 and 10 wpc. The second challenge experiment was otherwise performed under the same conditions as the first experiment. Both experiments were approved by the Norwegian Animal Research Authority and followed the European Union Directive 2010/63/EU for animal experiments.

### 2.7. RNA Isolation and Reverse Transcription Quantiative Polymerase Chain Reaction (RT-qPCR)

Total RNA was isolated from 20 µL heparinized blood homogenized in 650 µL QIAzol Lysis Reagent (Qiagen, Hilden, Germany) using 5 mm steel beads, TissueLyser II (Qiagen) and RNeasy Mini spin column (Qiagen) as recommended by the manufacturer. RNA was quantified using a NanoDrop, ND-1000 spectrophotometer (Thermo Fisher Scientific, Wilmington, DE, USA). The Qiagen OneStep kit (Qiagen) was used for RT-qPCR with a standard input of 100 ng (5 μL of 20 ng/μL) of the isolated total RNA per reaction in a total reaction volume of 12.5 µL. The template RNA was denaturated at 95 °C for 5 min prior to RT-qPCR targeting PRV gene segments S1, M2 and M3. The following conditions were used for S1: 400 nM primer, 300 nM probe, 400 nM dNTPs, 1.26 mM MgCl_2_, 1:100 RNase Out (Invitrogen) and 1 × ROX reference dye with the following cycle parameters: 30 min at 50 °C, 15 min at 94 °C, 40 cycles of 94 °C/15 s, 54 °C/30 s and 72 °C/15 s in an AriaMx (Agilent, Santa Clara, CA, USA). Similar conditions and cycle parameters were also used targeting M2 and M3, although primer concentration was adjusted to 600 nM and annealing temperature to 58 °C. All samples were run in duplicates, and a sample was defined as positive if both parallel samples had a Ct <35. The fluorescence threshold for S1, M2 and M3 was set at ΔRn 0.261, 0.028 and 0.021, respectively. The primers and probes are listed in Table S1. For analysis of antiviral gene expression, cDNA was prepared from 500 ng RNA using the QuantiTect reverse transcription kit with gDNA elimination (Qiagen) following the instructions from the manufacturer. Quantitative PCR was performed in triplets on 384-well plates using cDNA corresponding to 5 ng RNA in a total volume of 10 µL per parallel, SsoAdvanced™ Universal SYBR^®^ Green Supermix, and 500 nM forward and reverse primers (Table S2). The qPCRs were run for 40 cycles of 94 °C/15 s and 60 °C/30 s. All samples in the sample set were analyzed on the same plate using the same fluorescence threshold, and the cut-off value was set to Ct 37. The specificity of the SYBR green assays was confirmed by melting point analysis. Levels of Elongation factor (EF1α) mRNA were used for normalization of all assays by the ΔΔCt method.

### 2.8. Flow Cytometry

Samples consisting of 1.25 µL heparinized blood (diluted 1:20 in PBS) from each of the cohabitant fish in the first challenge experiment were plated into 96-well plates for intracellular staining as previously described [19] using anti-µNS and anti-ơ1 [4]. The corresponding zero serum, anti-µNS Zero and anti-σ1 Zero [4] were used as negative controls for background staining. Samples originating from 5 and 8 wpc were fixed, stained and analyzed immediately, while samples from 4 and 7 wpc were fixed and stored for one week and samples from 0, 3 and 6 wpc were fixed and stored for two weeks in flowbuffer (PBS, 1% BSA, 0.05% azide) before analysis. The cells were analyzed on a Gallios Flow Cytometer (Beckman Coulter, Miami, FL, USA), counting 50,000 cells per sample, and the data were analyzed using the Kaluza software (Becton Dickinson). Cells were gated according to size and granularity to include only intact cells and samples from 0 wpc were used as negative controls. Due to slight variation in background staining, the flow charts were gated individually to discriminate between negative and positive peaks.

### 2.9. Immunofluorescence Microscopy

Following flow cytometry analysis, the cells were prepared for immunofluorescence microscopy. The nuclei were stained with Hoechst trihydrochloride trihydrate (Life Technologies) and the cells were mounted to glass slides using Fluoroshield (Sigma-Aldrich, St. Louis, MO, USA) and cover slips. Images were captured on an inverted fluorescence microscope (Olympus IX81).

### 2.10. Transmission Electron Microscopy (TEM)

Samples consisting of 20 µL heparinized blood from each cohabitant fish in the first experimental challenge were diluted in 1 mL PBS, centrifuged at 1000× *g* for 5 min at 4 °C, washed twice in PBS and fixed in 3% glutaraldehyde overnight at 4 °C. All samples were further washed twice in PBS and prepared for transmission electron microscopy (TEM) as described earlier [19]. The sections were examined in a FEI MORGAGNI 268, and photographs were recorded using a VELETA camera.

### 2.11. Western Blotting (WB)

Heparinized blood from each cohabitant fish in the first challenge experiment was analyzed separately and as pooled samples from the different time-points. The samples were centrifuged at 5000× *g* and the blood pellets was lysed in Nonidet-P40 lysis buffer containing Complete ultra mini protease inhibitor cocktail and prepared for WB as described above. Anti-µNS (1:1000), anti-µ1C (1:500) [4], anti-σ1 (1:1000) [4], anti-σ3 (1:500) [2] and anti-λ1 (1:500) were used as primary antisera, Rabbit Anti-Actin (Sigma-Aldrich, St. Louis, MO, USA) was used to standardize the blots and Anti-Rabbit IgG-HRP (GE Healthcare) was used as secondary antibody. Blood collected at 0 wpc was used as negative control. In addition, heparinized blood from six of the cohabitant fish sampled at 0, 4, 6, 8 and 10 wpc in the second challenge experiment were prepared and analyzed in the same manner.

### 2.12. Immunoprecipitation (IP)

Blood from six cohabitants in the first challenge experiment sampled at 4, 5 and 8 wpc were pooled and lysed in Nonidet-P40 lysis buffer containing Complete ultra mini protease inhibitor cocktail as described above. The supernatants were transferred to new tubes and added anti-µNS or anti-µ1C (1:50) and incubated at 4 °C overnight with rotation. The Immunoprecipitation Kit Dynabeads Protein G (Novex, Life Technologies) was used for protein extraction and the beads were prepared according to the manufacturer’s protocol. The cell–lysate–antibody mixtures were mixed with the protein G-coated beads and incubated 2 h at 4 °C. The beads–antibody–protein complexes were washed according to the manufacturer’s protocol and run in SDS-PAGE. The SDS-gel was blotted onto PVDF membranes (Bio-Rad) and the proteins were detected using anti-µNS, anti-µ1C [4], anti-σ1 [4], anti-σ3 [2] and anti-λ1.

### 2.13. Liquid Chromatography–Mass Spectrometry (LC–MS)

Five and three fragments immunoprecipitated with anti-µNS (4 and 5 wpc) and anti-µ1C (5 wpc), respectively, that were not observed at 0 wpc, were excised and in-gel digested with 0.1 µg of trypsin in 20 µL of 50 mM ammonium bicarbonate, pH 7.8 for 16 h at 37 °C (Promega, Madison, WI, USA). The peptides were purified with µ-C18 ZipTips (Millipore, Billerica, MA, USA), and analyzed using an Ultimate 3000 nano-UHPLC system (Dionex, Sunnyvale, CA, USA) connected to a Q Exactive mass spectrometer (ThermoElectron, Bremen, Germany). Liquid chromatography and mass spectrometry was performed as previously described [37]. Data were acquired using Xcalibur v2.5.5 and raw files were processed to generate peak list in Mascot generic format (*.mgf) using ProteoWizard release (Version 3.0.331). Database searches were performed using Mascot (Version 2.4.0) against the protein sequences of λ1, λ2, λ3, µNS, µ1, µ2, σNS, σ1, σ2 and σ3 assuming the digestion enzyme trypsin and semi-trypsin, at a maximum of one missed cleavage site, fragment ion mass tolerance of 0.05 Da, parent ion tolerance of 10 ppm and oxidation of methionines, propionamidylation of cysteines, acetylation of the protein N-terminus as variable modifications. Scaffold 4.4.8 (Proteome Software Inc., Portland, OR, USA) was used to validate MS/MS based peptide and protein identifications.

### 2.14. Computational Analysis

Theoretical molecular weights for proteins were calculated using the Compute pI/Mw tool [38]. PSI-blast based secondary structure PREDiction (PSIPRED; Version 3.3) was used to predict protein secondary structure [39].

### 2.15. Statistical Analysis

Differences in gene expression levels of innate antiviral genes was analyzed using one-way Anova with Tukey’s multiple comparison test. Correlation analysis between PRV S1/M3 RNA levels and antiviral and immune gene expression were performed using nonparametric Spearman correlation.

## 3. Results

### 3.1. Viral RNA Load in Blood Cells

**Figure 1:**
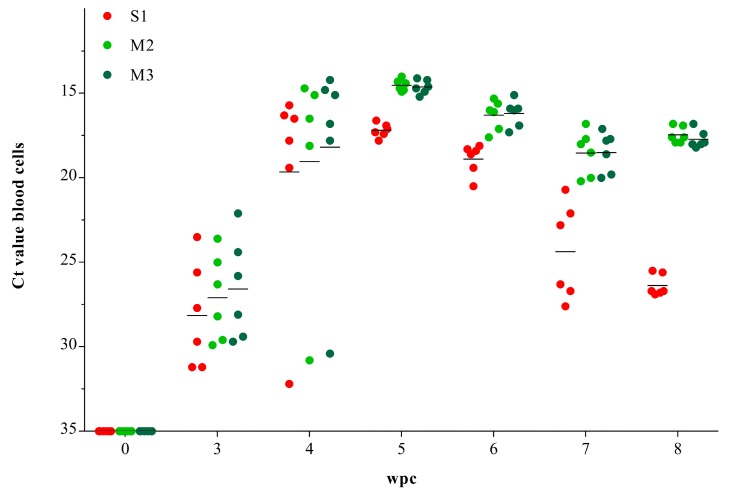
*Piscine orthoreovirus* (PRV) RNA load in blood cells. Reverse transcription quantitative polymerase chain reaction (RT-qPCR) of PRV gene segments S1, M2 and M3 in blood cells from cohabitant fish. Individual (**dots**) and mean (**line**) Ct-values, *n* = 6 per time-point. wpc = weeks post challenge.

RT-qPCR targeting PRV genomic segments S1, M2 and M3 revealed high viral RNA loads in blood cells from 3 to 8 wpc (Figure 1). RNA from segments S1, M2 and M3 were first detected at 3 wpc and peaked at 5 wpc with mean Ct-values of 17.2 (±0.4), 14.5 (±0.3) and 14.6 (±0.4). From 5 wpc, the S1 RNA load decreased, and by 8 wpc the mean Ct-value was 26.4 (±0.6). However, a similar decrease was not observed for the M2 and M3 RNAs, and by 8 wpc mean Ct-values for these genomic segments were 17.4 (±0.5) and 17.7 (±0.4), respectively. RT-qPCR targeting genomic segment S1 in blood from six fish sampled at 0, 4, 6, 8 and 10 wpc in the second challenge experiment was also performed and gave similar results (Figure S2).

### 3.2. Expression of Innate Antiviral Genes in PRV Infected Blood Cells

**Figure 2:**
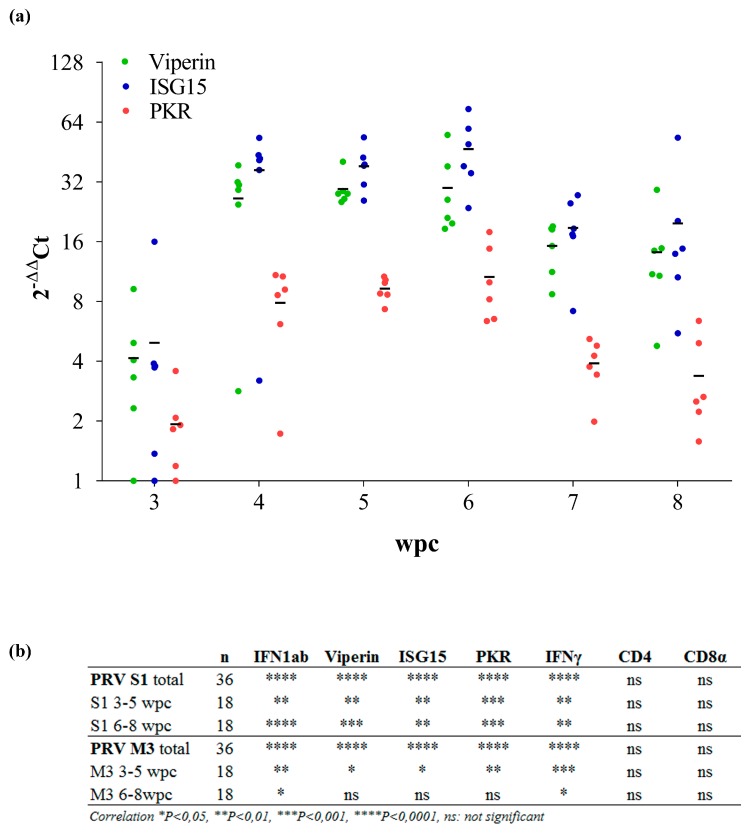
Expression of immune genes in blood cells. (**a**) Immune genes were assayed at 3–8 wpc by RT-qPCR in blood cells from cohabitant fish (*n* = 6 per time point). Data are normalized against EF1α and the lowest ΔCt level at 3 wpc (*n* = 6), and 2-ΔΔCt values are calculated. Mean relative expression is indicated. ISG = interferon-stimulated gene, PKR = double-stranded RNA (dsRNA)-activated protein kinase; (**b**) Correlation between Ct values for S1/M3 RNA and relative levels of antiviral gene expression for a set of immune genes.

The innate antiviral immune response in blood following PRV infection was studied by RT-qPCR targeting Atlantic salmon type I interferon (IFNab), viperin, interferon-stimulated gene 15 (ISG15), dsRNA-activated protein kinase (PKR) and IFNγ. All innate antiviral genes analyzed were statistically significantly upregulated during the peak phase of PRV infection from 4 to 6 wpc, increasing 5- to 20-fold compared to the level at 3 wpc (Figure 2a, Figure S3). The Ct values for S1 and M3 RNA correlated with the relative levels of gene expression for all innate antiviral genes, but not for the T-cell marker genes CD4 and CD8 (Figure 2b). When comparing the early phase up to the peak of infection (3–5 wpc) with the later phase (6–8 wpc), S1 RNA was correlated with the innate antiviral response in both phases, whereas M3 only showed significant correlation in the early phase (Figure 2b). EF1α were stably expressed during PRV infection and were used for normalization of all other assays by the ΔΔCt method (Figure S4) [15,40].

### 3.3. Flow Cytometry Indicates a Transient Peak in Blood Cells

**Figure 3:**
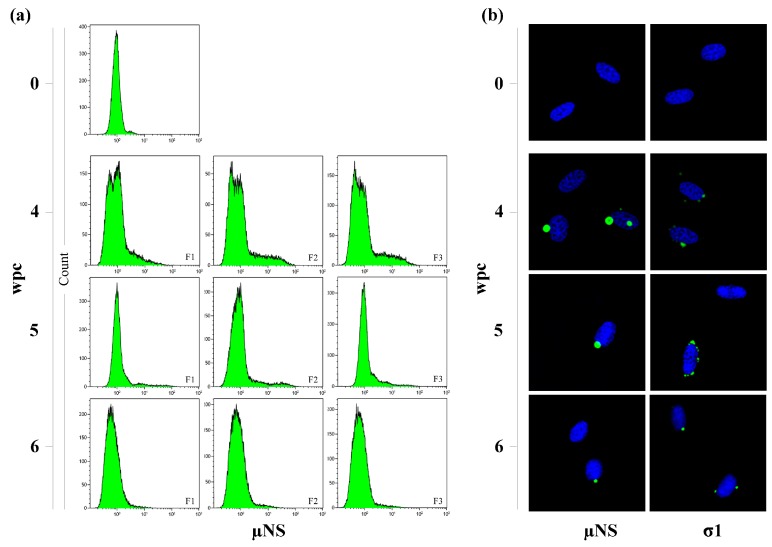
Presence of PRV µNS and σ1 in blood cells. (**a**) Intracellular staining of µNS in blood cells analyzed by flow cytometry from three cohabitant fish sampled at 4, 5 and 6 wpc. The negative control staining is one fish sampled at 0 wpc. A total of 50,000 cells were counted per sample and 30,000 were gated for analysis; (**b**) Fluorescent labeling of µNS (left) and σ1 (right) displaying viral factory-like inclusions (green) in infected red blood cells sampled 0 (negative control), 4, 5 and 6 wpc. The nuclei were stained with Hoechst (blue).

Blood cells stained intracellularly with anti-µNS and anti-σ1 were analyzed by flow cytometry (Figure 3a, Figure S5). A PRV positive population of blood cells was observed from 4 wpc as a marked shift in the histograms compared to negative samples. Five out of six fish were positive for µNS by flow cytometry at 4 wpc, consistent with the RT-qPCR data where the positive fish had lower Ct-values (18.2 ± 5.6) compared to the negative fish (30.4). At 5 wpc, the PRV positive blood cell population decreased, but was still visible for all individuals. From 6 wpc and onwards, no PRV-positive cell populations were observed. The pattern for σ1 positive cells was similar to that described for µNS.

### 3.4. Viral Factories Observed in Blood Cells

Both µNS and σ1 were detected by immunofluorescence as cytoplasmic globular inclusions in erythrocytes at 4, 5 and 6 wpc (Figure 3b). The inclusions varied in both size and number. At 4 and 5 wpc, they were predominantly large and perinuclear. Inclusions stained with anti-σ1 were generally smaller and more variable in size than those stained with anti-μNS. At 6 wpc, the number and size of the inclusions were considerably reduced and at 7 wpc and onward no inclusions were detected. These findings correlated with the results obtained from flow cytometry.

### 3.5. TEM of PRV Infected Blood Cells

**Figure 4:**
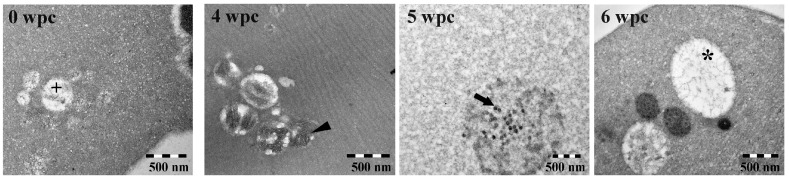
Transmission electron microscopy (TEM) of blood cells. PRV-infected red blood cells sampled at 0 (negative control), 4, 5 and 6 wpc show small empty vesicles (cross), lamellar structures (arrowhead), reovirus-like particles (arrow) and large empty inclusions (star).

TEM of PRV infected blood cells sampled at 0, 4, 5 and 6 wpc are shown in Figure 4. The control cells (0 wpc) contained circular cytoplasmic vesicles (200–500 nm) that were apparently devoid of specific content. In addition, a few control cells contained lamellar structures up to 300 nm in size. At 4 wpc, lamellar structures were frequent and a few large cytoplasmic inclusions (~800 nm) containing particles with reovirus-like morphology were observed. The viral particles were naked with an electron dense core that resembled previous TEM descriptions of PRV [19]. At 5 wpc, several small (200–500 nm) and large (~800 nm) cytoplasmic inclusions containing reovirus-like particles were detected. The larger inclusions contained a mixture of reovirus-like particles and lamellar structures, some enclosed within membrane-like structures. At 6 wpc, large inclusions were frequent, but only a few contained viral particles.

### 3.6. µNS Protein Expression in Individual Fish Correlate with viral RNA only during the Acute Phase of Infection

**Figure 5:**
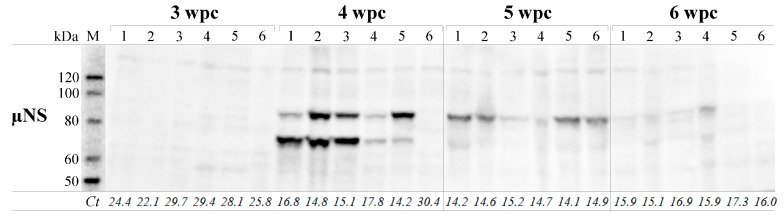
Detection of PRV uNS protein in blood cells compared to viral RNA load. Blood cells from 3, 4, 5 and 6 wpc (*n* = 6) analyzed for µNS by Western blotting. Ct-values for gene segment M3 (µNS) from the same samples are shown below each lane. M = molecular weight standard; Lane 1–6 refers to individual fish (1–6) per time point.

Blood cells from six fish sampled at 3, 4, 5 and 6 wpc were analyzed by WB using anti-µNS and compared to Ct-values targeting the corresponding genomic segment M3 of the same samples (Figure 5). No fish were positive by WB at 3 wpc, while five samples at 4 wpc demonstrated bands at molecular weight (MW) 83.5 (putative full-length µNS) and 70 kDa. The Ct-values from the same samples corresponded to the positive staining of the putative full-length µNS bands. Fish 6 at 4 wpc, was negative for µNS by WB; this individual also displayed a higher Ct-value (30.4) than the other cohabitants. The amount of µNS decreased markedly from 4 to 5 wpc, and the 70 kDa band was barely detectable at 5 wpc. At 6 wpc, the µNS protein was non-detectable by WB in fish 1, 5 and 6, and only barely detectable in the remaining fish. Although the µNS protein level decreased below the detection limit for WB, the corresponding viral RNA levels (genomic segment M3) remained high throughout the challenge. Thus, µNS protein and M3 RNA levels only correlated at 4 wpc.

### 3.7. PRV Protein Levels Display a Transient Peak in Blood Cells

**Figure 6:**
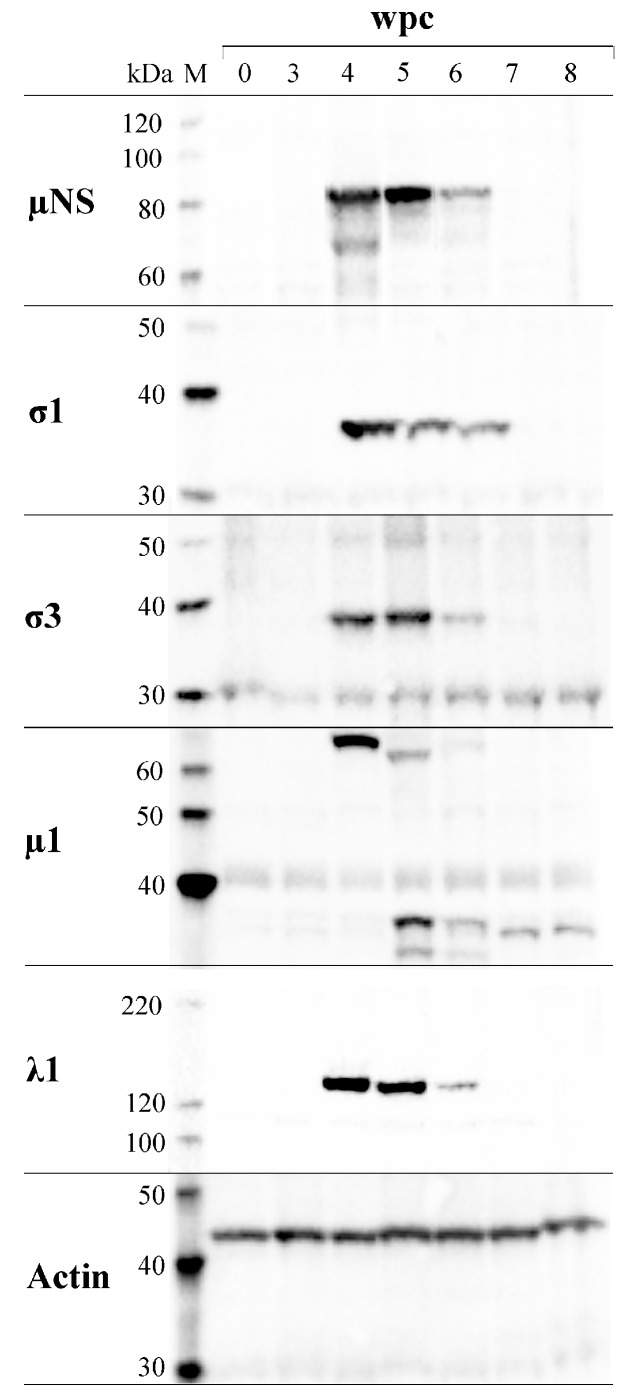
Presence of PRV proteins in blood cells. Pooled blood cell samples (*n* = 6) from each week were analyzed by Western blotting, targeting µNS, σ1, σ3, µ1 and λ1. M = molecular weight standard. Actin was used as control for protein load.

The load of structural proteins λ1, µ1, σ1 and σ3, and the non-structural protein µNS, displayed a similar transient peak at 4–6 wpc in blood cells (Figure 6). All five proteins appeared at 4 wpc and were non-detectable at 7 wpc. In addition to the putative full-length µNS, a band with the MW of about 70 kDa was observed at 4 wpc, consistent with findings from individual fish (Figure 5). The putative full-length µ1 protein (74.2 kDa) was detected at 4 wpc. However, at 5 wpc, this band was not present but replaced by three bands of approximately 70 kDa, 37 kDa and 32 kDa in size. At 7 and 8 wpc, only one band of approximately 35 kDa was detected. The same staining patterns for the λ1, µNS, µ1, σ1 and σ3 proteins were observed when blood from the second challenge experiment was analyzed (Figure S6).

### 3.8. PRV Proteins Interact with µNS

**Figure 7:**
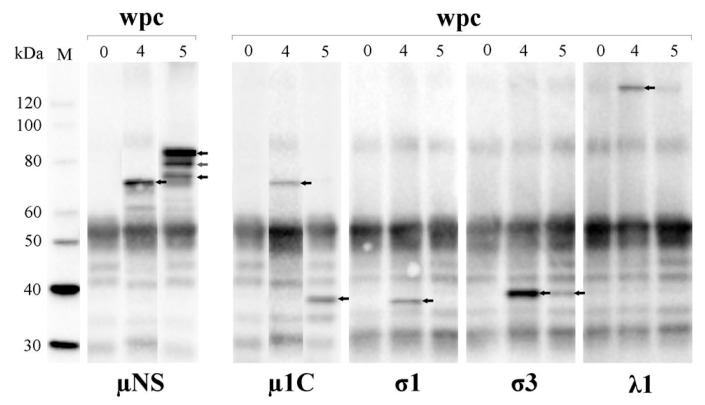
µNS interacts with multiple PRV proteins. Pooled blood cell lysate (*n* = 6) immunoprecipitated with µNS-antiserum, followed by Western blotting with primary antibodies detecting µNS, µ1C, σ1, σ3 and λ1 (arrows). M = molecular weight standard.

Interaction between µNS and other PRV proteins was studied by IP and WB (Figure 7). At 4 wpc, µNS was detected as a 70 kDa protein and at the same time-point the structural proteins λ1, µ1, σ1 and σ3 were co-immunoprecipitated. At 5 wpc, µNS was detected in three different sizes ranging from 70 kDa to 83.5 kDa (putative full-length µNS). However, the only structural proteins co-immunoprecipitating with µNS at 5 wpc were σ3 and the 35 kDa fragment of µ1 (see above). Interactions between µNS and other viral proteins were also investigated by liquid chromatography–mass spectrometry (LC–MS; Table 1) and peptides corresponding to λ1, λ2, λ3, µNS, µ1, σNS and σ1 were identified.

### 3.9. µNS Exists in Two Forms

WB of infected blood cells consistently produced two µNS bands of approximately 83.5 and 70 kDa (Figure 5 and Figure 6). Due to the presence of two translation initiation sites in MRV segment M3, the LC–MS data were analyzed to identify putative shorter variants of the PRV µNS. The peptide distribution along the full-length µNS sequence and their spectrum matches are shown in Figure S7a. The µNS peptides and total spectrum matches obtained from the two bands are shown in Figure S7b. Several N-terminal µNS peptides were identified from the 83.5 kDa band that were not observed in the 70 kDa band. Furthermore, the peptide spectrum matches from the 83.5 kDa and 70 kDa bands in the 200 amino acid N-terminus were 10 to 1, respectively. In contrast, for the remaining C-terminal µNS sequence, the 83.5 kDa and 70 kDa bands produced similar or identical peptide spectrum matches, with a ratio of 66 to 63 (Figure S7). These results point to the presence of a second translation initiation site in the 5’- region of the µNS ORF. Start sites at M_85_, M_94_, M_115_ or M_169_ would provide proteins with predicted sizes of 74.5, 73.6, 71.1 and 65.5 kDa, respectively. M_115_ is the most likely candidate due to its size and presence in all PRV strains.

### 3.10. µ1 Has Two Putative Proteolytic Cleavage Sites

WB targeting the µ1 protein showed that the protein is present in different forms during infection. The putative full-length µ1 (74.1 kDa) was detected at 4 wpc (Figure 6 and Figure 7). In contrast, smaller versions, with estimated sizes of 70, 37 and 32 kDa, replaced the full-length variant at 5 wpc (Figure 6). The three size variants from 5 wpc were subjected to LC–MS analysis (Figure S8). The 70 kDa band most likely represents µ1C following pre-cleavage at N_42_P_43_ (MW 69.8 kDa). Of the fourteen peptide spectrum matches identified from the 70 kDa band, two were found to overlap N_42_P_43_ (Figure S8a). This is most likely due to carryover of slightly larger full-length μ1 (74.1 kDa) following gel excision. No peptides stretching N-terminal to N_42_P_43_ were identified from the 37 and 32 kDa bands. Additional semi-tryptic peptides, i.e., peptides generated by trypsin cleavage at one end but not the other, were identified from both the 37 and 32 kDa bands (Figure S8a). Among these is a peptide identified from the 32 kDa band harboring an N-terminal S_388_. Cleavage of µ1C at F_387_S_388_ would yield N- and C-terminal fragments of 37.7 kDa and 32.1 kDa, respectively. The distribution of peptide sequences and peptide spectrum matches provides support for proteolytic cleavage at or close to F_387_S_388_ (Figure S8). The results suggest that the 37 kDa and 32 kDa bands represent the PRV homologues of MRV µ1 fragments δ and φ, respectively. Besides the µ1 peptide sequences, peptides originating from other PRV proteins with sizes close to the sizes of the three excised fragments, were also identified. Peptide sequences matching λ1 and µNS (one peptide spectrum match each) were identified from the 70 kDa band, sequences matching σ1, σ3 and σNS were identified from the 37 kDa band (2, 2 and 11 peptide spectrum matches, respectively) and σ2 sequences were identified from the 32 kDa band (four peptide spectrum matches).

## 4. Discussion

Screening of farmed Atlantic salmon has indicated that PRV is ubiquitous in seawater and causes a persistent infection [9,11,41,42]. The study of PRV pathogenesis has been hampered by the lack of susceptible cell lines, and is currently dependent upon in vivo experiments. The fish in this experiment were challenged by cohabitation, i.e., through a natural transmission route. To ensure coordinated onset of infection, a high ratio of shedder fish was used. We found that PRV infection of salmon blood cells is acute and transient, with a peak lasting for 1–2 weeks under these experimental conditions.

Erythrocytes are major target cells for PRV [19]. Piscine erythrocytes are nucleated and contain the transcriptional and translational machinery enabling virus replication both in vivo and ex vivo [16,19]. We detected various PRV proteins in blood cells from 4 wpc, and the amount of protein was reduced at 6 wpc. Innate antiviral gene expression also peaked at 4–6 wpc and all selected genes were significantly induced during the peak period, in line with PRV protein production. In contrast to the transient peak displayed by PRV proteins, the viral RNA levels in blood cells persisted. The viral RNA level though, varied for the targeted genomic segments; the level of M2 (µ1) and M3 (µNS) remained high throughout the trial, while S1 (σ3) transcripts decreased from 6 wpc. TEM analysis corresponded well with viral protein production, i.e., the lamellar structures observed at 4 wpc developed into inclusions containing reovirus-like particles at 5 wpc, while no virus particles could be observed at 7 wpc. The findings support PRV, causing an acute infection in blood cells where high PRV protein and particle production are sustained 1–2 weeks before the infection becomes persistent. Our study shows that, after the acute phase, the PRV RNA level as determined by RT-qPCR does not reflect the virus load in blood.

The salmon does not appear to be able to eliminate PRV. Challenge experiments have shown that PRV RNA can be detected at a steady level in heart and liver until 36 wpc (end of experiment) [41], and in blood for more than a year after challenge [9,19]. In an experiment where the infectious potential of persistently PRV infected Atlantic salmon was studied, sentinel fish were added at 59 wpc, but no transmission to the sentinel fish was observed [9]. This indicates that fish persistently infected with PRV do not continuously shed the virus. Viral persistence is common in fish and has been demonstrated for several RNA viruses [9,43,44,45,46,47]. The only PRV protein that could be detected after the peak of virus protein production was a fragment of µ1, suggesting a possible role for this protein in persistent infection. In farmed salmon, where the size of the population in a net pen may exceed a hundred thousand individuals, and in the whole farm be more than a million fish, viral persistence in the population, but not necessarily in the individual, is also a critical parameter.

PRV infection in erythrocytes has previously been shown to induce expression of type I interferon and interferon-regulated genes [16,18]. In this study, the level of viral RNA correlated with the innate antiviral response in individual fish, with the exception of M3 expression after the virus peak (6–8 wpc). The continuous production of M3 RNA indicates that the innate antiviral immune response primarily inhibits virus replication post transcriptionally, which is in line with the functions of PKR and ISG15 on translation and protein modification, respectively [48,49].

Orthoreoviruses generate viral factories in the cytoplasm of infected cells [21,27,50,51,52], and PRV forms cytoplasmic globular viral factories resembling the structures produced by MRV T3D [16,19,31]. Viral factories are structures where virus replication and assembly occur, and thus where the viral proteins co-localize. The secluded nature of the viral factories modulates the level of the innate antiviral immune response. The orthoreoviral protein µNS is orchestrating the construction of the factories and in this study and earlier studies we have found that λ1, λ2, λ3, µ1, σNS, σ1, σ2 and σ3 interact with µNS [31]. The σ3 protein co-precipitated with µNS but was not identified by MS, however WB can be more sensitive than LC–MS [52]. This suggests that µNS interacts directly or indirectly with all three λ-proteins, the µ1 protein, and possibly all four σ-proteins. The µNS protein was detected in different molecular sizes at specific time points. Further investigations led to the finding of four possible internal translation initiation sites in the µNS gene. The M_115_ residue was determined to be the best candidate as M_94_ is not conserved among all PRV isolates, and M_85_ and M_169_ are unlikely due to the sizes of the proteins generated. Post-translational cleavage to generate µNSC as shown for ARV µNS cannot be excluded, although the specific proteolytic cleavage site in the ARV protein is not conserved in PRV [2,33,53]. The different µNS size variants, i.e., full-length µNS and the 70 kDa variant with putative translation initiation at M_115_, may differ in their interactions with other PRV proteins. At 4 wpc, when only the 70 kDa variant of µNS was detected following IP, all targeted structural proteins co-precipitated. However, at 5 wpc, when full-length µNS was dominant, only the σ3 protein and the assumed µ1 fragment δ co-precipitated. Studies previously performed on aquareoviruses and ARV indicate that recruitment of viral proteins into viral factories occurs in a predefined order through direct or indirect association with µNS [50,54].

Four different molecular sizes of the µ1 protein were observed in the infected blood cells. Previous multiple sequence alignments of the µ1 amino acid sequence showed absolute conservation of the G2-myristoylation site and the autolytic N_42_P_43_ cleavage site, both regarded as crucial for reovirus µ1-mediated membrane penetration [2]. The band observed at 4 wpc represents the full-length µ1 protein while the 70 kDa band at 5 wpc most likely represents µ1C.

Although peptides containing amino acid sequences overlapping the N_42_P_43_ site were observed from the 70 kDa band following LC–MS, peptides ending in P_43_ were present in equal amount. We conclude that the presence of the N_42_P_43_ overlapping peptides originate from carryover of the slightly larger full-length µ1 following gel excision. In addition, proteins can exhibit different abilities to separate in SDS-PAGE. This explains the presence of a minor fraction of peptides from the δ fragment (i.e., 37 kDa band) in the φ fragment (i.e., 32 kDa band) and vice versa. No peptide sequences overlapping N_42_P_43_ were identified from the 37 and 32 kDa bands. Rather, a higher number of peptides with an N-terminal P_43_ generated by non-tryptic cleavage were identified, providing additional support for cleavage at N_42_P_43_.

MRV µ1 contains a second cleavage site in its C-terminal region which, upon cleavage by exogenous proteases, generates the additional fragments δ and φ [55]. In the present study, we propose that the 37 kDa and 32 kDa bands represent the PRV homologues of the MRV δ and φ proteins. Hence, PRV φ contains a larger N-terminal portion of µ1 compared to MRV φ. Although there is only 28% identity at the amino acid level [2], the secondary structure of the PRV µ1 monomer predicted by PSIPRED [39] (not shown) is very similar to that of MRV µl [56]. This includes the helix-rich region in the C-terminal end [57], which for MRV largely constitutes the φ fragment shown to be crucial for membrane penetration, apoptosis induction and intracellular localization [57,58]. An interesting observation is that the three PRV µ1 peptide sequences detected in the 35 kDa band following IP with anti-µNS were all N-terminal to the proposed φ region, suggesting that µNS-interacting sites on µ1 may be located in the proposed δ region, between P_43_ and F_387_. From 7 wpc and onwards, the only PRV protein detected was a ~35 kDa protein which could represent the δ proteolytic fragment.

Production-related diseases are often multifactorial and the outcome of a PRV infection is influenced by viral strain, age of the fish, production and environmental factors. Recently, PRV was demonstrated to be the etiologic agent of EIBS, causing anemia and mass mortality in juvenile Coho salmon [7]. The level of anemia in EIBS corresponded well with the level of viral replication in blood and it is therefore tempting to suggest that EIBS is a consequence of acute PRV infection, i.e., the direct effect of virus PRV replication in erythrocytes. PRV is also the causative agent of HSMI [1,4], which appears 2–3 weeks after virus replication peaks in blood cells. The dominance of CD8 positive inflammatory cells found in the HSMI specific heart lesions indicates that immune mediated mechanisms are a major cause of the myocarditis.

In this study, we show that PRV infection has an acute phase in blood cells with high virus production before the infection subsides to a low persistent level. The continued transcription of viral RNA in the persistent phase suggests that the innate antiviral immune response may act to inhibit the virus infection post transcriptionally.

## Figures and Tables

**Table 1:** Identified *piscine orthoreovirus* (PRV) peptides following immunoprecipitation with anti-µNS and mass spectrometry (MS).

* Band Excised from SDS-PAGE (kDa)	Identified PRV Proteins	Unique Peptides	Theoretical PRV Protein Size (kDa)
140 (5 wpc)	µNS	1	83.5
130 (5 wpc)	λ3	2	144.5
	λ2	7	143.7
	λ1	14	141.5
	µNS	9	83.5
	σ1	1	34.6
80 (5 wpc)	λ1	11	141.5
	µNS	24	83.5
70 (4 wpc)	µNS	16	83.5
35 (5 wpc)	µNS	4	83.5
	δ ^†^	3	37.7
	σNS	1	39.1
	σ1	2	34.6

* Approximate size of proteins excised from bands following IP with anti-μNS antisera at four and five weeks post challenge (wpc). ^†^ Proteolytic fragment of μ1 proposed in the present work.

## Supplementary Materials

The following are available online at www.mdpi.com/1999-4915/9/3/49/s1, Figure S1: Specificity of µNS and λ1 antisera; Figure S2: PRV RNA load in blood cells (second challenge experiment); Figure S3: Expression of immune genes in blood cells; Figure S4: Expression of EF1α during PRV infection; Figure S5: PRV σ1 positive blood cells detected by flow cytometry; Figure S6: Presence of PRV proteins in blood cells (second challenge experiment); Figure S7: LC–MS analyses of PRV µNS; Figure S8: LC–MS analyses of PRV µ1; Table S1: Primers and probes used for construction of plasmids and expression of viral RNA levels; Table S2: Primers used for analysis of antiviral gene expression.
