# Supplementary material for: Viral Protein Kinetics of Piscine Orthoreovirus Infection in Atlantic Salmon Blood Cells
Source: Viruses. 2017 Mar 18;9(3):49. doi: 10.3390/v9030049 (PMC5371804; doi:10.3390/v9030049)
Supplement: Supplementary file 1 [file viruses-09-00049-s001.pdf]

Supplementary Materials: Viral Protein Kinetics of Piscine Orthoreovirus Infection in Atlantic Salmon Blood Cells

Hanne Merethe Haatveit, Øystein Wessel, Turhan Markussen, Morten Lund, Bernd Thiede, Ingvild Berg Nyman, Stine Braaen, Maria Krudtaa Dahle and Espen Rimstad

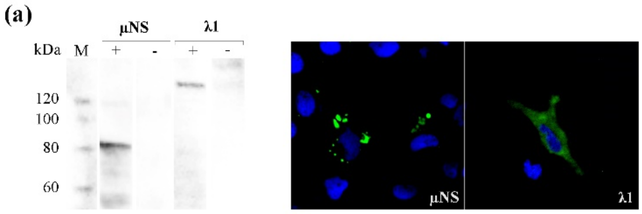

Figure S1. Specificity of µNS and λ1 antisera. (**a**) The specificity of the µNS and λ1 antisera were verified by western blotting of lysates from EPC cells transfected with plasmids encoding µNS or λ1, or mock transfected. Anti-rabbit IgG HRP conjugate was used as secondary antibody. M = molecular weight standard. The theoretical MWs of µNS and λ1 are 83.5 and 141.5 kDa, respectively. (**b**) Staining of µNS (left) and λ1 (right) in transfected EPC cells using rabbit IgG conjugated with Alexa Fluor 488 (green) as secondary antibody. The nuclei were stained with Hoechst (blue).

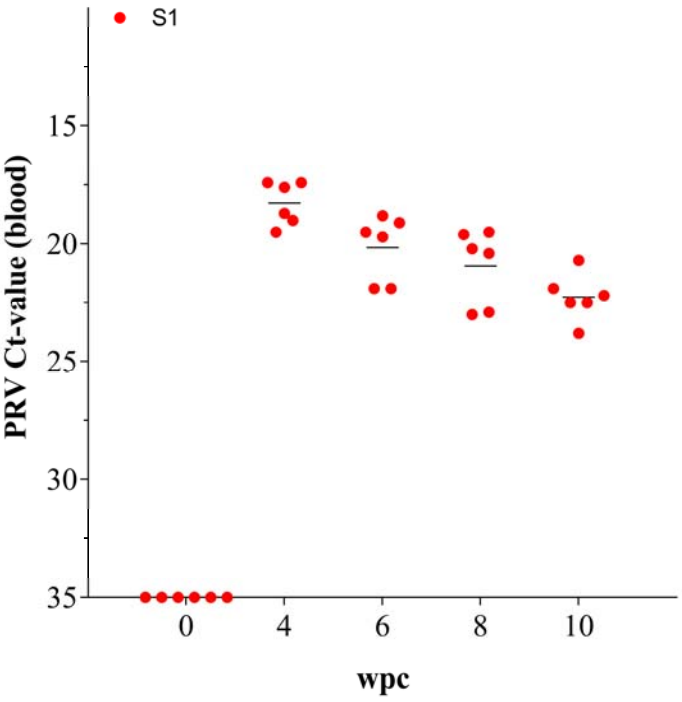

Figure S2. PRV RNA load in blood cells (second challenge experiment). RT-qPCR of PRV gene segment S1 in blood cells from cohabitant fish. Individual (dots) and mean (line) Ct-values, n = 6 per time-point. wpc = weeks post challenge.

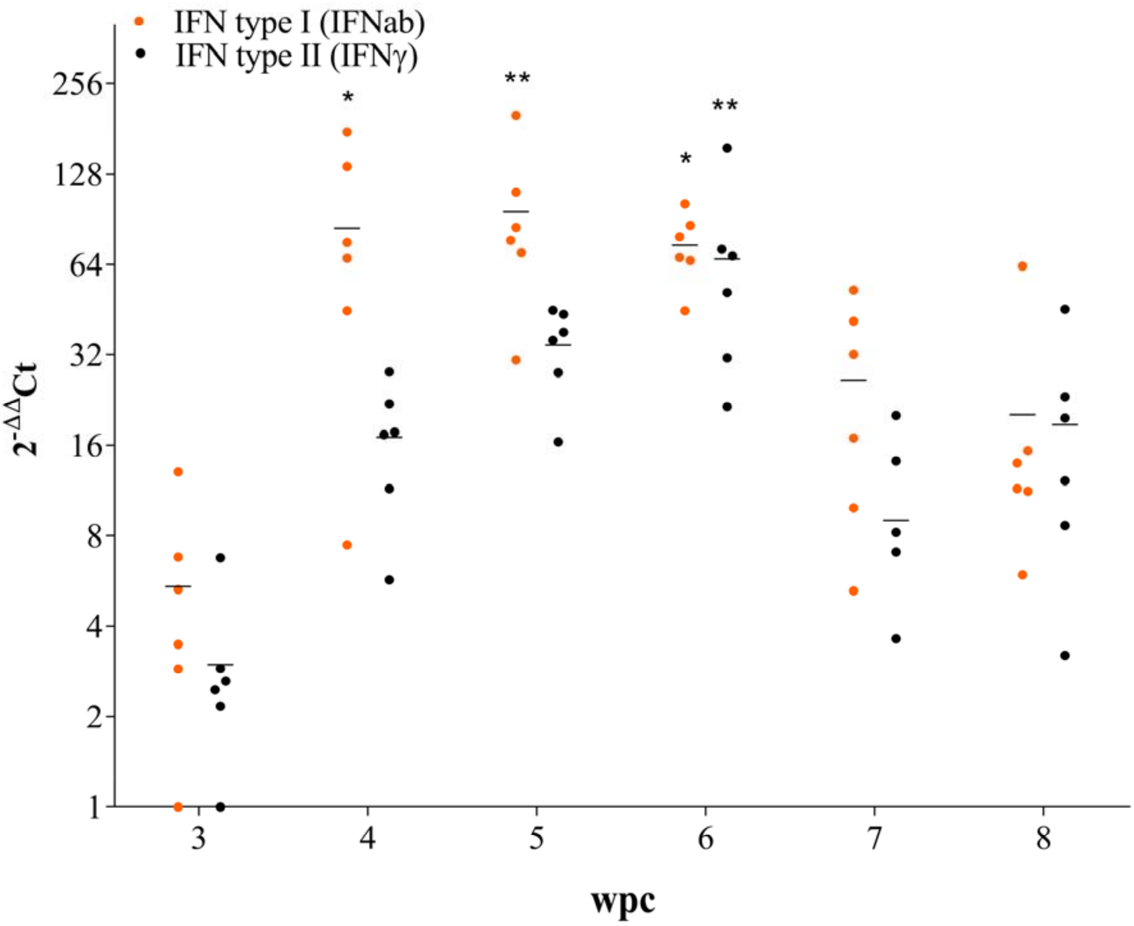

Figure S3. Expression of immune genes in blood cells. Immune genes were assayed at 3-8 wpc by RT-qPCR in blood cells from cohabitant fish (n= 6 per time point). Data are normalized against EF1α and the mean ΔCt level of each target at 3 wpc (n=6), and 2-ΔΔCt values are calculated. Relative expression +/- SD is shown. wpc = weeks post challenge, IFN = interferon.

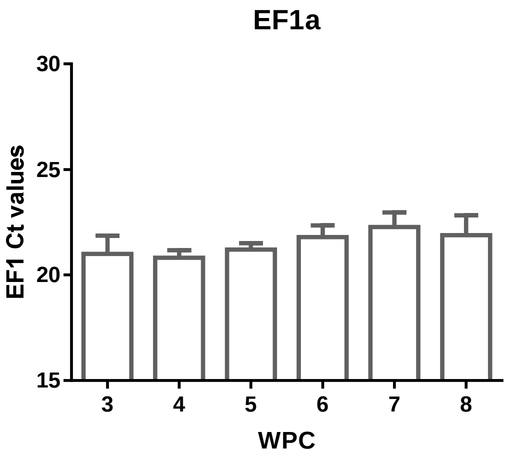

Figure S4. Expression of EF1α during PRV infection.

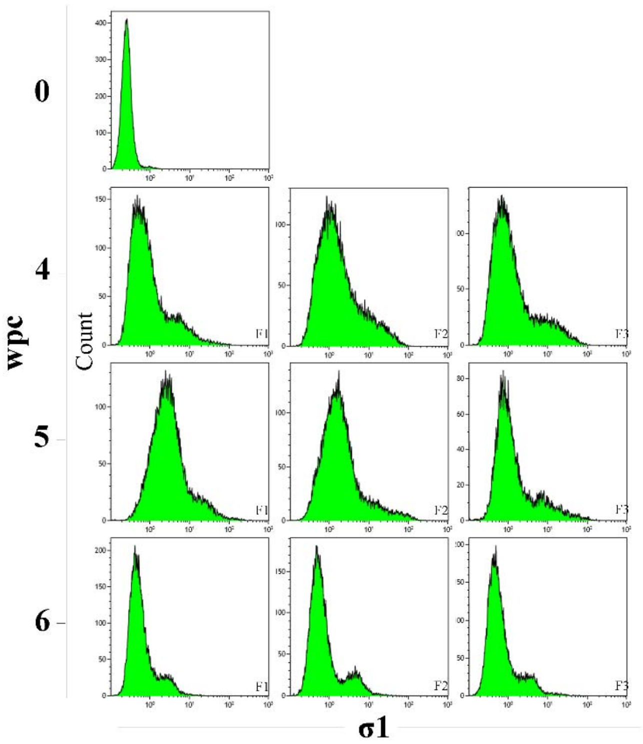

Figure S5. PRV σ1 positive blood cells detected by flow cytometry**.** Flow cytometry result showing intracellular staining of σ1 in blood cells from three cohabitant fish sampled 4, 5 and 6 wpc. One fish sampled 0 wpc is presented as negative control. 50 000 cells were counted for each sample and 30 000 cells were gated for analysis.

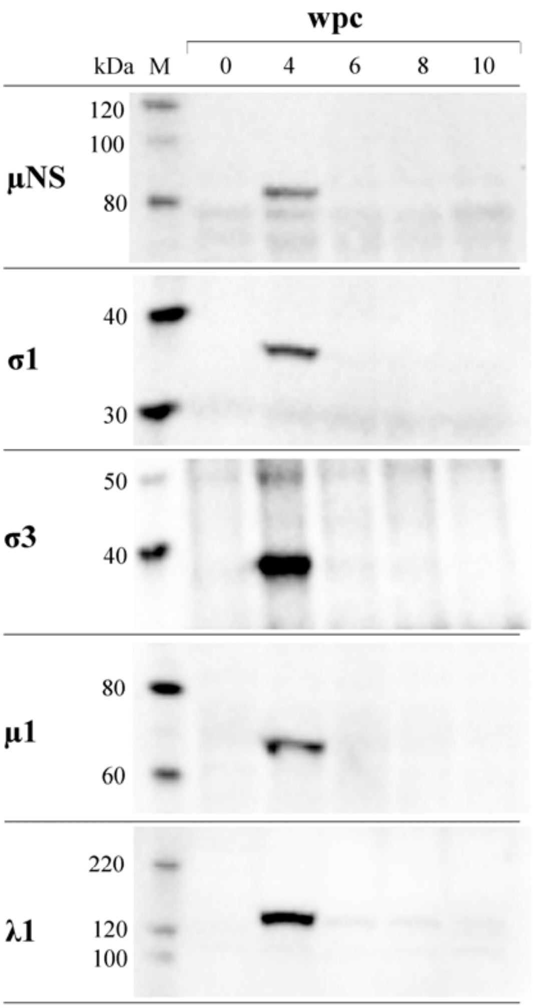

Figure S6. Presence of PRV proteins in blood cells (second challenge experiment). Pooled blood cell samples (n = 6) from each week were analyzed by western blotting targeting μNS, σ1, σ3, μ1 and λ1. M = molecular weight standard, wpc = weeks post challenge.

(a)

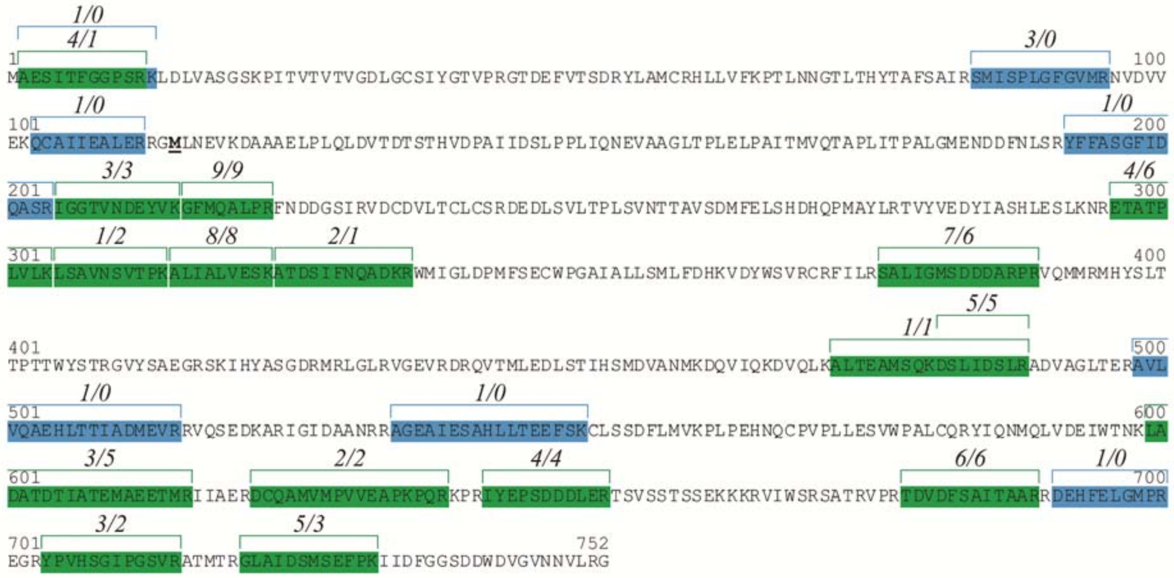

(b)

Band excised from SDS-PAGE (kDa)	Unique µNS peptides N-terminal	Unique µNS peptides C-terminal
83.5	10	66
70	1	63

Figure S7. LC-MS analyses of PRV µNS. (**a**) PRV µNS peptide sequences identified following IP with anti-µNS antisera, SDS-PAGE, excision of the 70 kDa (4 wpc) and 83.5 kDa (5 wpc) fragments, tryptic digestion and LC-MS analysis. Peptides identified from the 83.5 kDa fragment are shown with blue background, while peptides derived from both fragments are with green background. Peptides exclusive to the 70 kDa band were not identified. Numbers in italic indicate peptide spectrum matches derived from both bands, i.e. in the order 83.5 kDa/70kDa. The putative secondary in-frame translation initiation site encoding M_{115} is shown in bold and underlined. (**b**) PRV µNS peptide spectrum matches identified in the 200 aa N- and the 552 aa C-terminal regions of µNS, derived from the 70 kDa and 83.5 kDa bands.

(a)

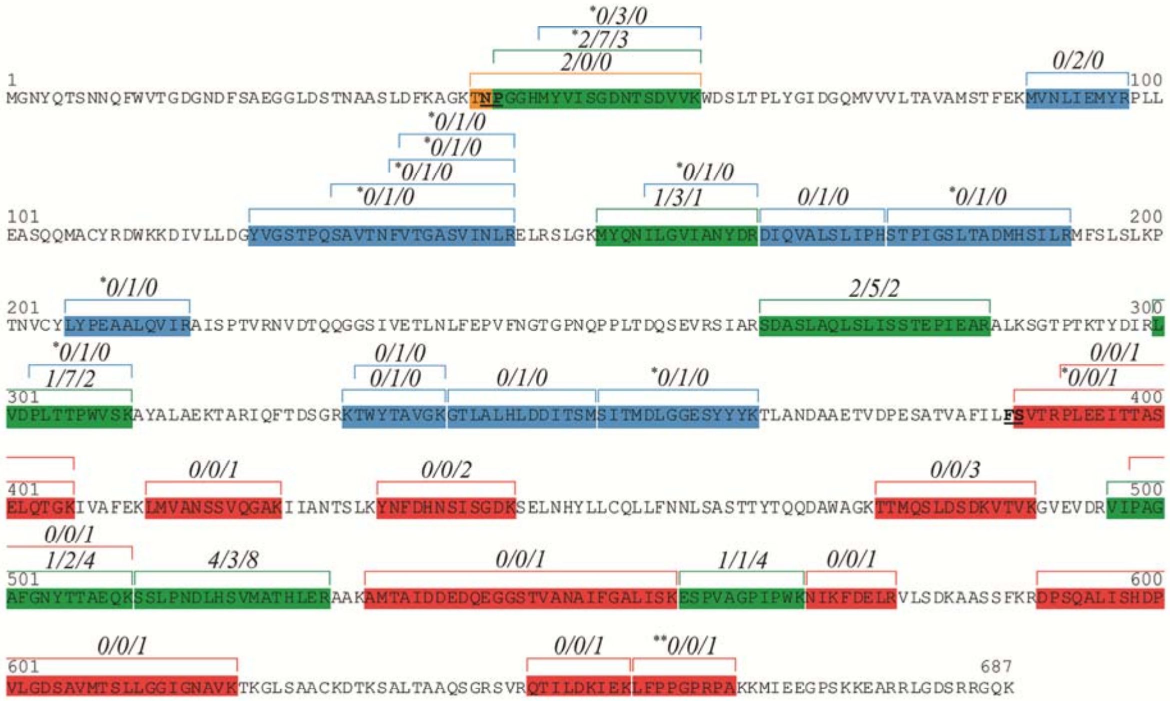

(b)

Band excised from SDS-PAGE (kDa)	Unique µ1 peptides N-terminal to S_{388}	Unique µ1 peptides C-terminal to F_{387}
70	8	6
37	40	8
32	6	30

Figure S8. LC-MS analyses of PRV µ1. (**a**) PRV µ1 peptide sequences identified following IP with anti-µ1C antisera 5 wpc, SDS-PAGE, excision of the 70 kDa, 37 kDa and 32 kDa fragments, tryptic digestion and LC-MS analysis, shown with orange, blue and red backgrounds, respectively. Green background indicate peptides identified from all three fragments (common peptide sequences from two bands only were not observed). The cleavage site $N_{42}P_{43}$ and the putative cleavage site at $F_{387}S_{388}$ are shown in bold and underlined. Numbers in italic indicate peptide spectrum matches derived from the three bands, i.e. in the order 70kDa/37kDa/32kDa. *Non-tryptic N-terminal cleavage site. **Non-tryptic C-terminal cleavage site. (**b**) PRV µ1 peptide spectrum matches N- and C-terminal to $S_{388}F_{387}$ identified from the 70, 37 and 32 kDa bands.

Table S1. Primers and probes used for construction of plasmids and expression of viral RNA levels.

Plasmid/target	Primer sequence (5′ → 3′)	
pENTR μNS	Fwd	CACC**ATG**GCTGAATCAATTACTTTTGGAGGA
	Rev	GCCACGTAGCACATTATTCACGCCCAC
pET100 λ1	Fwd	CACC**ATG**GAGCGACTTAAGAGGAA
	Rev	TTAGTTGAGTACAGGATGAGTCAA
S1	Fwd:	GCGTCCTGCGTATGGCACC
	Rev:	GGCTGGCAT GCCCGAATAGCA
	Probe (FAM):	ATCACAACGCCTACCT MGBNFQ
M2	Fwd:	CCCTCAAGCCAACCAATGTGTGCTA
	Rev:	GTGTCAACATTCCTGACAGTCGGTGAA
	Probe (FAM):	TAGCTCGAATCACTTGCAG MGBNFQ
M3	Fwd:	GTCATCTGGAGTCGTTCTGCCACT
	Rev:	GCACACTGCCAGGAATCCCACT
	Probe (FAM):	TTCATCACGTCTTGCG MGBNFQ

Table S2. Primers used for analysis of antiviral gene expression.

Target gene	Primer/probe sequence (5′-3′)	Genbank acc. no.
EF1α	Fwd: TGCCCCTCCAGGATGTCTAC<br>Rev: CACGGCCCACAGGTACTG	BG933897
IFNab	Fwd: CCTTTCCCTGCTGGACCA<br>Rev: TGTCTGTAAAGGGATGTTGGGAAAA	DQ354152
Mx	Fwd: GATGCTGCACCTCAAGTCCTATTA<br>Rev: CACCAGGTAGCGGATCACCAT	XM_014133086
Viperin	Fwd: AGCAATGGCAGCATGATCAG<br>Rev: TGGTTGGTGTCCTCGTCAAAG	NM_001124253
ISG15	Fwd: GGCCTGCATTCAGGATCTAA<br>Rev: TACAGTCTCACCAGGCACCA	AY795563
ssPKR	Fwd: CAGGATGCAACACCATCATC<br>Rev: GGTCTTGACCGGTGACATCT	EF523422
IFNγ	Fwd: AAGGGCTGTGATGTGTTTCTG<br>Rev: TGTACTGAGCGGCATTACTCC	NM_001171804
CD8α	Fwd: GTCTACAGCTGTGCATCAATCAA<br>Rev: GGCTGTGGTCATTGGTGTAGT	NM_001123583
CD4	Fwd GCCCCTGAAGTCCAACGAC<br>Rev: AGGCTTCTCTCACTGCGTCC	NM_001146408

© 2017 by the authors. Licensee MDPI, Basel, Switzerland. This article is an open access article distributed under the terms and conditions of the Creative Commons by Attribution (CC-BY) license (http://creativecommons.org/licenses/by/4.0/).
